# Building bridges: how women’s relational empowerment is linked to well-being and community embeddedness

**DOI:** 10.3389/fsoc.2024.1466161

**Published:** 2024-10-31

**Authors:** Hazem Aldabbas, Liza Gernal, Ahmed Zain Elabdin Ahmed, Abdallah M. Elamin

**Affiliations:** University of Science and Technology of Fujairah, Fujairah, United Arab Emirates

**Keywords:** empowerment of women, relational empowerment, well-being, community embeddedness, United Arab Emirates

## Abstract

Both host nations and expatriates themselves are concerned with the integration of women expatriates into host countries’ societies. We developed a framework based on empowerment theory to illustrate how relational empowerment influences well-being and community embeddedness in a host country. By promoting relational empowerment, individuals enhance their well-being, making them more engaged and embedded within a community. This study collected data from 218 women expatriates living in the United Arab Emirates (UAE) and utilized the Hayes PROCESS Macro to test four proposed hypotheses. Based on bootstrapping and regression results, we found that women’s relational empowerment is directly and indirectly linked to community embeddedness, with this indirect relationship influenced by factors such as women’s well-being. We discussed the implications of these findings for both theoretical advancement and the development of practical strategies, emphasizing on how relational empowerment can impact women’s well-being and lead to greater community embeddedness in the UAE and potentially elsewhere.

## 1 Introduction

The empowerment of women is seen as a crucial requirement for human and sustainable development, in line with the United Nations’ sustainability goals outlined in the UN 2030 agenda (Venugopalan et al., 2021). The term “empowerment” refers to a complex social process that enables individuals to exert control over their lives, communities, and societies. It allows them to take action on issues they deem important (Page and Czuba, 1999). In the past two decades, empowerment has emerged as a central focus of global development initiatives (Upadhyay et al., 2014). This study contends that there is a pressing need for a critical examination of existing literature on women’s studies, coupled with empirical validation. Women’s empowerment has garnered significant attention from scholars, practitioners, and various non-governmental organizations (NGOs), particularly in emerging and developing nations. Additionally, women’s empowerment is a fundamental human rights issue, as it is pivotal in protecting human dignity and social justice (Samier and ElKaleh, 2021).

In the context of this study, community embeddedness is understood as the perceived worth of community-related benefits lost by departing a destination country (Stahl et al., 2024) and demonstrates an individual’s commitment and dedication to their surrounding community (Chen and Shaffer, 2017). Put simply, community embeddedness describes a psychological phenomenon that motivates individuals to remain in a certain situation or environment (Singh et al., 2018). Community embeddedness likewise describes one’s connection to the social web of one’s residential area (Lee et al., 2004). Hence, community embeddedness describes individuals’ connection to their communities beyond the confines of their work environments.

Social psychological interactional theory explores the role of self-perception, cognitive processes, and social attitudes within various group contexts (Hogg, 2016). In this study, community embeddedness is framed as an outcome variable. Similarly, Schaubroeck et al. (2022) theorized community embeddedness as an outcome. The authors found that perceived insider status and organizational identification, operationalized as antecedent variables, correlated positively with community embeddedness.

Empowerment is conceptualized through multiple dimensions, such as meaning, self-determination, competence, and impact, all of which are pertinent to work environments (Hassard et al., 2022; Spreitzer, 1995). Psychological empowerment is viewed as the integration of personal control over beliefs, a proactive approach to enhancing one’s life, and a more nuanced understanding of cultural contexts (Mushtaq and Mehmood, 2023). Good mental health is a crucial aspect of women’s well-being; therefore, to achieve full empowerment, psychological empowerment is required (Moubarak et al., 2022). To synthesize these definitions, the psychological empowerment of women is defined as the presence of a set of psychological states essential for individuals to perceive control over their involvement (Spreitzer, 2008). Additionally, this study adopts the conceptualization of relational empowerment as proposed by Peterson et al. (2021). Women’s empowerment, thus encompasses emotional, cognitive, behavioral, and relational dimensions, making it a multidimensional concept (Peterson et al., 2021). Therefore, understanding women’s empowerment as relational empowerment could have implications for the enhancement of cognitive empowerment, as individuals engage in active listening, reflection, and facilitation of cognitive changes in others (Christens, 2012).

Studying women’s empowerment is crucial because it is a factor that significantly affects women’s well-being. Examining the mediating role of well-being among women expatriates represents a vital area of research. Well-being, in this context, encompasses various aspects of individuals’ lives deemed essential by both them and their communities, and they are crucial for the realization of their full potential (Lewis and Rödlach, 2019). This study delves into the well-being of women expatriates, aiming to provide valuable insights into their experiences and the factors influencing their overall sense of well-being. Higher levels of positive well-being are associated with better health, including higher levels of positive feelings toward life in general (Aldabbas and Bettayeb, 2024). In the context of initiating, nurturing, and expanding entrepreneurial endeavors, well-being is understood as the overall experience of satisfaction, positive emotions, limited occurrence of negative emotions, and psychological functionality (Wiklund et al., 2019).

An expatriate is broadly defined as someone who has moved from their home country to reside or work in another country for an extended period (Vance and McNulty, 2014). In this study, a woman expatriate is defined as an individual who has independently chosen to move abroad in search of better job opportunities or improved life circumstances. Additionally, this work focuses on the community embeddedness of women expatriates in the United Arab Emirates (UAE) because this group faces unique challenges, such as having to navigate traditional gender roles and balance professional and personal responsibilities. The cultural context of a host country also significantly affects the level of pressure expatriates experience, particularly due to strong expectations surrounding gender roles and family responsibilities (Tahir, 2023). Understanding women expatriates’ integration into local communities is essential in addressing gender-specific challenges and enhancing support systems. This integration has ultimately contributed to discussions on diversity, inclusion, and expatriation in global labor markets. These challenges can influence women expatriates’ professional success, community integration, and overall well-being, making their experiences distinct from those of their male counterparts.

**Figure 1:**
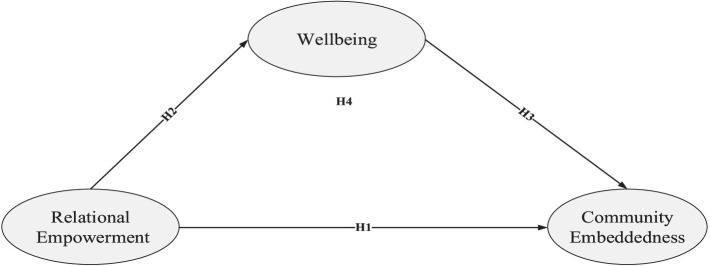
Hypothetical framework.

For the past three decades, expatriate research has primarily concentrated on expatriate success, emphasizing their adjustment and withdrawal (Chen and Shaffer, 2017). However, the lives and work experiences of expatriates are increasingly attracting academic interest due to the fast-paced changes in the nature and structure of work within global labor markets (Rodriguez and Ridgway, 2019). Thus, ensuring the inclusion of women in a diverse country like the UAE presents a challenge. This is primarily because the UAE is categorized as being among the most challenging destinations for global assignments (Tahir and Savara, 2019). The focus of this investigation is the UAE, a nation with a substantial expatriate population, rendering it an exemplary case study (Hassk-Saheem et al., 2022). According to UN estimates, the country’s population reached 10 million by 2023, with expatriates accounting for 88.1% of this figure [Central Intelligence Agency (CIA), 2024]. Moreover, this study responds to many calls in the literature to examine relational empowerment (Christens, 2012; Rodrigues et al., 2018). Specifically, expatriate research has not fully elucidated the factors influencing women expatriates’ well-being and its impact on expatriates living and working in the UAE (Haak-Saheem, 2016). Therefore, it is essential to understand how the relational empowerment of employees is related to community embeddedness via well-being (see Figure 1).

To the best of our knowledge, this is the first study to examine the mediating role of well-being in the relationship between relational empowerment and community embeddedness. Additionally, the findings contribute to the broader literature by enhancing our understanding of expatriate studies. By exploring the relational empowerment, well-being, and community embeddedness of women expatriates, this research fills a gap in the literature on the UAE context and provides insights into how gender impacts expatriation experiences. The implications of this research can inform policies and support systems for women expatriates, promoting diversity and inclusion in the UAE and potentially elsewhere, considering the UAE is one such country where the workforce is predominantly made up of expatriates (Tahir and Savara, 2019). As such, this study focuses on addressing the following research questions:

Is there a direct relationship between women’s relational empowerment and community embeddedness? If so, does well-being mediate the relationship between women’s relational empowerment and community embeddedness?

## 2 Literature review and hypotheses development

The research posits that empowerment theory offers the most suitable framework for comprehending the connection between individual well-being and the broader social context, as proposed by Perkins and Zimmerman (1995). As a result of significant research attention directed toward empowerment over the past four decades, empowerment has evolved into a foundational concept in the realm of social work (Joseph, 2020). Additionally, this study distinguishes itself from previous research on women’s empowerment by addressing all categories of women expatriates—employed, unemployed, or retired—to provide more comprehensive insights into the concept of relational empowerment in fostering well-being and community embeddedness.

The social context for women expatriates in the UAE is shaped by the country’s diverse cultural environment (Harrison and Michailova, 2012). While the UAE is generally open and welcoming to foreign professionals, women expatriates may encounter unique challenges, such as having to navigate traditional gender roles, adjusting to cultural norms, and balancing work and family responsibilities. These factors can sometimes limit their participation in their communities, as cultural expectations might place greater pressure on women to fulfil their social and family obligations. Meanwhile, the UAE’s diverse population, including large expatriate communities, offers well-being support and a multicultural environment (Universal Relocations, 2023). For instance, self-initiated expatriates frequently compare locations and favor the UAE for its strong reputation, perceiving it as offering more benefits than other countries (Arifa et al., 2021). Additionally, Emirati culture emphasizes family unity, respect, conservatism, hospitality, and Islamic values, which expatriates are encouraged to respect (Universal Relocations, 2023).

The UAE’s large expatriate community is primarily engaged in professional roles, often forming social circles within their own groups while seeking to integrate with locals. Their average stays can range from several months to years. According to an annual global survey by Cigna, expatriates who relocate to the UAE tend to stay longer compared to those who move to other countries, with the average expat stay in the UAE being 4.4 years, significantly exceeding the global average of 3.2 years (Gokulan, 2022).

Among the problems that expatriates face, the gender wage gap is one that the UAE government has actively attempted to tackle through legislative measures, notably with the enactment of Federal Decree Law No. 6 of 2020 (WAM, 2020). This law mandates equal pay for men and women performing jobs of equal value in the private sector (TimeCamp, 2024). Despite these legal advancements, a gender wage gap persists, with variations across different sectors and job roles. On average, individuals in the UAE earn between $30,000 and $55,000 annually (TimeCamp, 2024). Next, we discuss the four hypotheses.

### 2.1 The relationship between relational empowerment and community embeddedness

Community embeddedness refers to the extent of individuals’ participation and involvement in their local communities, which encompass social networks, shared activities, and collective experiences. This measure quantifies the depth and breadth of connections individuals maintain within their communities, significantly influencing their sense of belonging and attachment to them. Women’s empowerment is likely affected by prevalent behaviors and norms informed by national, organizational, and project cultures (Aldabbas et al., 2021), which impact the formation of community embeddedness. Thus, to encourage empowerment at the community level, policymakers should encourage joint endeavors to ensure access to governmental and other communal resources (Perkins and Zimmerman, 1995). Research has shown that women’s empowerment, especially in relational circumstances, has a significant impact on a number of areas of women’s life, including community embeddedness. Notably, the presence of social support has been found to positively influence both community identification and embeddedness (Chiu et al., 2019).

Factors within a society’s structure, such as gender roles and cultural norms, can either facilitate or hinder women’s agency within their relationships (Kabeer, 1999). In collectivist societies like Vietnam, entrenched gender norms may constrain women’s decision-making abilities, particularly in matters concerning finances (Huis et al., 2020). Additionally, research has highlighted the complex dimensions of women’s empowerment, such as relationships, societal dynamics, and individual autonomy (Huis et al., 2020). Specifically, women’s independence and self-governance are significantly influenced by relational empowerment, which entails possessing decision-making power within intimate relationships (Huis et al., 2020).

Studies have revealed a positive association between empowerment, including its relational aspect, and women’s sense of community embeddedness (Aldabbas et al., 2024). Moreover, the link between women’s empowerment and community facilitators underscores the vital role community embeddedness has played in promoting women’s agency and well-being (Huis et al., 2017). Therefore, relational empowerment highlights how social norms are shaped due to community embeddedness (Vander Plaat, 1999).

Empowerment is an evolving process that encompasses several interconnected dimensions, including resources (which involve access to and future entitlements to material, human, and social assets) and agency (which refers to the ability to set and pursue personal goals) (Costa et al., 2023). This comprehensive approach contributes significantly to enhancing women’s trust and relational empowerment, leading to greater community interaction and engagement. Both empowerment and community embeddedness are crucial for individuals’ achievements and welfare across various fields. Research has highlighted the correlation between organizational and community embeddedness, emphasizing that high levels of community embeddedness enhance organizational embeddedness and work outcomes by providing access to valuable resources and support (Ng and Feldman, 2013). Moreover, empowerment is often supported by external experiences of participation in one’s environment, where individuals build a sense of community and commitment (Mouchrek and Benson, 2023). We therefore expect a positive relationship between women’s empowerment and community embeddedness, resulting in the first hypothesis:

H1. Women’s relational empowerment is positively related to women’s community embeddedness.

### 2.2 The relationship between relational empowerment and well-being

Women’s empowerment is not a necessary result of economic strength; instead, it involves recognizing the ideology that legitimizes male domination and understanding how it perpetuates women’s oppression. Empowerment is conceptualized as a spiral, changing consciousness, identifying target areas for change, planning strategies, action, and analysis (Mosedale, 2005). Idris et al. (2023) support the notion that women’s relational empowerment positively affects their well-being. It includes aspects like influence, connection, and autonomy in interpersonal relationships. Women are more inclined to stand up for their rights, express their opinions, and make life-changing decisions when they feel empowered in their relationships (Idris et al., 2023). Gender disparities in well-being are more pronounced in nations with greater gender inequality, less financial growth, and stronger adherence to sexist gender norms (Zhu and Chang, 2020).

Relationships that women build in their marriages, families, and communities have a dynamic impact on women’s relational empowerment and well-being. These connections can boost women’s empowerment and general well-being by giving them access to resources, growth opportunities, and support. Women who experience higher levels of empowerment (e.g., relational empowerment) are more capable of advocating for themselves and their families, consequently enhancing the overall well-being of their households. Eluwole et al.’s (2022) empirical research found that empowerment correlates with improved well-being. Furthermore, a study involving 541 women and men revealed that holding a managerial position had a more significant influence on inequalities in well-being and satisfaction than gender itself (Frederick and Lazzara, 2020).

In the context of this study, there is a favorable correlation between women’s relational empowerment and their well-being. By understanding these dynamics and their significance for women’s empowerment, we may effectively advance gender equality and improve women’s well-being. From this context, we formulated our second hypothesis as follows:

H2. Women’s relational empowerment is positively related to women’s well-being.

### 2.3 The relationship between well-being and community embeddedness

Academics and psychologists characterize “good life” as comprising genuine self-expression, a feeling of well-being, and active participation in life and work (Sutton, 2020). Less frequent participation in activities associated with cultural characteristics, such as visiting museums or historical sites, is positively correlated with satisfaction (Wheatley and Bickerton, 2017). Thus, empowered women who are part of robust and encouraging social networks are more likely to experience improved well-being and an overall higher quality of life (Aldabbas et al., 2024).

Community well-being depends on various factors working together to improve the quality of life for everyone in a community. For instance, encouraging older adults to engage in lifelong learning can greatly enhance community well-being (Merriam and Kee, 2014). An exploratory study revealed and validated the strong correlation between community engagement and personal factors such as self-esteem, as well as knowledge and skills in linguistic and contextual domains, contributing to the enhancement of overall well-being (Alfieri et al., 2019).

A study of 105 respondents revealed a significantly high level of work spirituality and job embeddedness among participants (Rajappan et al., 2017). Drawing from a diverse sample of 165 employees and coworkers, another study observed that social support from both organizations and communities correlated positively with embeddedness in various domains (Singh et al., 2018). With this in mind, we formulated our third hypothesis as follows:

H3. Women’s well-being is positively related to community embeddedness.

### 2.4 The mediating role of well-being

The well-being of women expatriates in the UAE has been minimally explored in the literature. Hassk-Saheem et al. (2022) investigated the well-being of “low-status” expatriates in the UAE, while other studies focused on the relationship between empowerment and well-being (Annan et al., 2021; Estevan-Reina et al., 2021; Marin-Garcia and Bonavia, 2021). However, limited attention has been devoted to the relational empowerment of women expatriates.

Since the 1980s, research has consistently shown that income is the strongest predictor of whether a woman will leave or stay in an abusive relationship. Other predictors include being married and experiencing less severe intimate partner violence (IPV). Studies show that women who have been physically abused by their partners consistently self-report their health as poor compared to women who have no experience of IPV. They are also more likely to have worse health behaviors, such as smoking tobacco and drinking alcohol, having a poor diet, and using more pain medications (Wong and Mellor, 2014).

The nature of employment, spousal support, and coping styles have been studied in relation to well-being. For instance, marital status, the quality of the marital relationship, and spousal support are important determinants of well-being (Rao et al., 2003). Additionally, job and social network-related variables, together with coping styles, emerged as significant contributors to well-being (Rao et al., 2003).

As stated earlier, women’s empowerment is a multifaceted concept that encompasses various dimensions, including personal empowerment, relational empowerment, and collective empowerment (Huis et al., 2017). A study conducted in India showed that skill development, treatment by employers, compensation for injuries, monthly savings, and number of people in a household significantly impact the overall well-being of domestic workers (Gupta, 2022).

The social well-being of domestic workers is significantly influenced by their treatment by employers, opportunities for skill development, compensation for injuries, and health benefits. Economic health is determined by income, savings, and monthly expenses. Women in the informal economy often come from socially and economically disadvantaged groups, making it difficult to secure employment. Additionally, the COVID-19 pandemic forced many to return to their hometowns. Therefore, further research is needed to develop policy strategies to change society’s perspective on domestic work (Gupta, 2022). Given that women’s relational empowerment is closely linked to their community embeddedness and overall well-being, we formulated our mediator hypothesis as follows:

H4. The relationship between women’s relational empowerment and community embeddedness is mediated by women’s well-being.

## 3 Research methodology

The objective of this study was to examine the impact of well-being on the association between relational empowerment and community embeddedness. To address this objective, a quantitative investigation employing multivariate regression was conducted to evaluate the proposed conceptual framework.

### 3.1 Sample and data collection

A survey was distributed via an online link to a cohort of women residents in the UAE by one of the authors who had access to large women groups. The findings derived from this research will advance the knowledge of the difficulties as well as the potentials that usher women leaders in their respective careers. The link was shared within these groups, resulting in the selection of 218 respondents between December 2023 and January 2024. Specifically, participants were selected based on their availability and willingness to participate. The sample primarily consisted of university students from classes that one of the researchers was teaching at the time, as well as individuals from various groups within which the authors were working (e.g., university or industry). WhatsApp and especially LinkedIn’s integration, as well as the use of email for the distribution of the mentioned questionnaire, and the concentration on C-Suite learners in the Chartered Management Institute (CMI) and Chartered Management program (CMP), enable a thorough data collection process. Digital media played a significant role in distributing the questionnaire to women learners across various emirates in the country.

Women empowerment is not a new policy in the country. It is quite innovative that apart from focusing on the important matter of women in leadership positions, it also ensures that the government itself remains supportive to the women empowerment programs around the country. Additionally, some participants came from communities in different emirates. Presently the federal government through various policies and the implementation of the programs has made considerable progress on the mainstreaming of gender equality as well as women’s empowerment despite the different emirates to which they come from. This study revealed the existence of shared values, rules, policies, advocacies, and practices of women expatriates in the region. One of them is to create a separate web resource for online services designed exclusively for women. This site offers materials, data, and services that are designed to promote women’s positions in society and in careers. These groups were easily accessible to the researchers, and participation was voluntary. Convenience sampling was chosen due to time constraints and the exploratory nature of the research (Etikan et al., 2016). In the survey, we could not reach all key participants involved in model development. Thus, we utilized convenience sampling (Ozkaya and Akdur, 2021), a method commonly used when researchers have limited control over the selection process. The sample size was calculated for multiple regression analysis (G*Power), aiming for an effect size (f^2^) of 5%, a power of 95%, and a margin of error of 5%. The calculation indicated that a sample of 218 respondents would be required considering the two predictor variables.

Table 1 breaks down the demographics of the study participants. The largest group comprising 23.9% of the study population was 32–37-year-olds, followed by the 38–43 group at 21.6%. The CMI and CMP attract a majority of C-Suite participants. This is the reason why the majority of the age group ranges from 32–43 years old, as enrollment requires individuals holding executive positions. This targeted demographic ensures that the data collected reflects the insights and experiences of high-level professionals. Most of the participants had at minimum a bachelor’s degree (53.7%), followed by a master’s degree (19.7%). Married individuals accounted for 58.7% of the study population, followed by single individuals at 32.6%. Most of the cohort was employed and working full-time at 71.6%, followed by students at 11.0%. Participants who had lived in the UAE for 1 to 4 years comprised the largest group (22.5%), followed by those who had lived in the UAE for more than 21 years (20.6%). The countries of origin for the women in the study are broken down as follows: the largest group, representing 28%, was from India, followed by women from the Philippines at 24%. Participants from Jordan accounted for 11%, while those from Syria represented 6%. Egypt made up 5% of the study population, with Sudan, South Africa, and Lebanon each contributing 3%. Canada accounted for 2%, and a diverse group from other countries collectively comprised 17%.”

**Table 1:** Demographic variables.

Demographic variables	Frequency	Percent
Age		
18–25 years	34	15.6
26–31 years	25	11.5
32–37 years	52	23.9
38–43 years	47	21.6
44–49 years	34	15.6
50 and above	26	11.9
Education level		
High school	13	6.0
Bachelor’s	117	53.7
Master’s	43	19.7
Doctoral studies	40	18.3
Other	5	2.3
Marital status		
Single	71	32.6
Married	128	58.7
Widowed	2	0.9
Divorced	11	5.0
Separated	6	2.8
Employment status		
Employed, working full-time	156	71.6
Employed, working part-time	12	5.5
Not employed, looking for work	16	7.3
Not employed, not looking for work	5	2.3
Student	24	11.0
Retired	3	1.4
Others (e.g., business owner)	2	0.9
Number of years living in the UAE		
1–4 years	49	22.5
5–8 years	33	15.1
9–12 years	39	17.9
13–16 years	30	13.8
17–20 years	22	10.1
Above 21	45	20.6
Country of origin		
India	60	28
Philippines	52	24
Jordan	23	11
Syria	12	6
Egypt	10	5
Sudan	6	3
South Africa	6	3
Lebanon	6	3
Canada	5	2
Other (Algeria, USA, UK, German, China, Iran, etc.)	38	17
Total	218	100.0

### 3.2 Measurements

#### 3.2.1 Relational empowerment

The construct, comprising nine items, was derived from Peterson et al. (2021) (see Appendix). The Cronbach’s alpha for the nine items was 0.894. Responses were recorded on a Likert scale ranging from “Almost never” to “Almost always”.

#### 3.2.2 Well-being

In this research, the five-item World Health Organization Well-Being Index (WHO-5) was used. It is a concise and universally applicable scale designed to assess subjective well-being, and it comprises only positively formulated items (Topp et al., 2015) (see Appendix). The questions were designed to measure the extent of positive emotions experienced over the past two weeks. Responses to the WHO-5 items were recorded using a six-point Likert scale, ranging from 0 (“At no time”) to 5 (“All of the time”). For the current sample, the Cronbach’s alpha was *α* = 0.854.

#### 3.2.3 Community embeddedness

Community embeddedness was measured using a nine-item scale outlined by Felps et al. (2009) (see Appendix). Of the original pool of 21 items, this research specifically selected nine items focusing on involvement in community activities, following Schaubroeck et al. (2022). Additionally, embeddedness was often conceptualized at the individual level following the framework of Chen and Shaffer (2017). It is noteworthy that the reliability coefficient of the Cronbach’s alpha for the scale used in this study was 0.842. The Likert scale used to measure this construct ranged from “Strongly disagree” to “Strongly agree.”

## 4 Results

### 4.1 Descriptive statistics

Table 2 presents the means, standard deviations (Std), correlations among the variables, skewness, and kurtosis. The correlation between well-being and community embeddedness was notably strong (r = 0.428) and significant at the 1% level. Relational empowerment and community embeddedness exhibited the second-highest correlation (r = 0.354) that was also significant at the 1% level. Furthermore, there was a positive and significant correlation at 1% between relational empowerment and well-being (r = 0.343). It is noteworthy that our sample ranged within ±1.96.

**Table 2:** Correlations (r), mean, standard deviation, skewness, and kurtosis.

Variables	RE	WB	CE	Mean	Std. deviation	Skewness	Kurtosis
RE	1			5.359	1.036	−0.700	0.386
WB	0.343**	1		2.842	1.059	−0.282	−0.683
CE	0.354**	0.428**	1	5.162	1.294	−0.735	0.367

**Correlation was significant at the 0.01 level (two-tailed). RE: relational empowerment; WB: well-being; CE: community embeddedness.

### 4.2 Common method bias test

Common method bias (CMB) was addressed to ensure the reliability of the research findings. Since the data were collected from a single source, CMB could potentially have been an issue, we applied Harman’s single-factor test (Podsakoff et al., 2003). The analysis, using an unrotated factor method, revealed that the first factor explained 31.728% of the variance. Since this value was below the 50% threshold, it confirmed that CMB did not pose a significant issue in our study.

### 4.3 Confirmatory factor analysis

Confirmatory factor analysis (CFA) of the hypothesized model showed that it fit well. Specifically, a comparative fit index (CFI) of 0.933, which exceeded the 0.90 threshold, indicated strong model fit. Hu and Bentler (1999) suggested that a Tucker–Lewis index (TLI) of 0.920, approaching 0.950, represents a satisfactory result. The study’s standardized root mean square residual (SRMR) was 0.072, well below the 0.08 benchmark, signifying a good fit. Additionally, the root mean square error of approximation (RMSEA) was 0.061, falling under the 0.08 limit, and was therefore considered reasonable error, as supported by Chen et al. (2008). Thus, all survey items showed acceptable fit (Table 3). Furthermore, alternative models did not show a good fit. For example, merging relational empowerment and well-being resulted in a CFI of 0.714, TLI of 0.672, SRMR of 0.124, and RMSEA of 0.124, indicating poor fit. Similarly, when all factors were merged, the results indicated a poor fit (CFI = 0.459, TLI = 0.405, SRMR = 0.143, and RMSEA = 0.167). These findings confirm that, from a statistical perspective, our hypothesized model fit best, reinforcing our argument that women’s relational empowerment is linked to well-being and community embeddedness.

**Table 3:** Comparison of alternative models.

Model	X^2^	df	CFI	TLI	SRMR	RMSE
Three factors (hypothesized model)	381.255	212	0.933	0.920	0.072	0.061
Two factors (merging of RE and WB)	947.121	221	0.714	0.672	0.124	0.124
One factor (merging of RE, WB, and CE)	1600.567	230	0.459	0.405	0.143	0.167

RE: Relational empowerment; WB: Well-being; CE: Community embeddedness.

### 4.4 Hypotheses tests

This study used SPSS, specifically Hayes’ PROCESS macro, employing a 95% confidence interval and 5,000 bootstrap iterations. The aim was to examine the potential mediating role of well-being in the relationship between relational empowerment and community embeddedness, following the framework proposed by Hayes (2018). We used this specific statistical method because it relies on ordinary least squares (OLS) regression, which is a widely employed technique for linear statistical models (Hayes and Cai, 2007). Following Certo et al. (2016), we used OLS as an estimator to address potential endogeneity issues without introducing bias into our sample. Employing the PROCESS macro for mediation analysis has also become standard practice in the social sciences (Sarstedt et al., 2020). Furthermore, OLS measures the effect size (beta) of independent variable(s) *x* on dependent variable *y* in an unbiased and effective manner (Certo et al., 2016).

To examine our conceptual model, we started with the first hypothesis on the direct association between relational empowerment and community embeddedness, revealing a positive and statistically significant relationship (B = 0.293, t = 3.687, *p* < 0.001). Hypothesis two was supported by the direct association observed between relational empowerment and well-being, revealing a positive and statistically significant relationship (B = 0.351, t = 5.373, *p* < 0.001). Hypothesis three was supported by the direct association observed between well-being and community embeddedness, revealing a positive and statistically significant relationship (B = 0.424, t = 5.460, *p* < 0.001). With regard to the last hypothesis, the mediation analysis indicated that the relationship between relational empowerment and community embeddedness was mediated by well-being (point estimate: 0.149, 95% CI: 0.077–0.239). Thus, the indirect relationship was found to be positive and significant (B = 0.149) at the 5% significance level (see Table 4).

**Table 4:** Regression results.

Well-being (mediator)							Community embeddedness (Dependent Variable)					
	B	SE	t	*p*-value	LLCI	ULCI	B	SE	t	*p*-value	LLCI	ULCI
RE	0.351	0.065	5.373	0.000	0.222	0.480	0.293	0.079	3.687	0.000	0.136	0.450
WB	–	–	–	–	–	–	0.424	0.078	5.460	0.000	0.271	0.578
Constant	0.961	0.357	2.694	0.008	0.258	1.661	2.385	0.414	5.759	0.000	1.569	3.202
R^2^ = 0.118							R^2^ = 0.232					
*F* (1,216) = 28.869 < 001							*F* (2,215) = 32.435 < 001					

*N = 218; B = Beta; SE = Standard error; LLCI = Lower level of confidence interval; ULCI = Upper level of confidence interval; RE = Relational empowerment; WB = Well-being; CE = Community embeddedness.*

## 5 Discussion and implications

This study examined the direct relationship between the relational empowerment of women expatriates and their community embeddedness (Hypothesis 1). The literature suggests that empowerment, including relational empowerment linked to community embeddedness, is underpinned by various factors such as social support, psychological well-being, and organizational effectiveness (Aldabbas et al., 2024; Chiu et al., 2019; Huis et al., 2017). We also examined the relationship between relational empowerment and well-being (Hypothesis 2). The findings disclosed a positive and significant association supported by prior research (e.g., Ahmed and Malik, 2019; Omang et al., 2022; Rawat, 2014). Empowering women is not only purely a critical objective but also a means to grant them the authority to drive significant progress in economic endeavors, positioning them as equal contributors alongside their male counterparts (Al-Hassan and Al-Dulaimi, 2023). This will thus help empower women and bolster their autonomy, ultimately leading to an increase in their level of satisfaction and well-being. The findings of this study align with a study conducted in the UAE that suggested that adaptive capacity, including empowerment and coping ability, may provide the essential resources to maintain higher levels of happiness, even when confronted with challenging life conditions such as immigration-related rearrangements and life adjustments (Mendonca et al., 2020).

With regard to Hypothesis 3, we examined well-being and community embeddedness. Recent studies have found that well-being is related to community embeddedness (Merriam and Kee, 2014; Singh et al., 2018). When a woman feels that she is active and fulfilled by things that interest her, these feelings are expected to have a positive effect on her personal life and community embeddedness. In relation to Hypothesis 4, we examined the mediating role of well-being in the relationship between relational empowerment and community embeddedness. Few studies have examined these constructs together. A prior study revealed the noteworthy and positive mediating role of well-being in the relationship between digitalization and mental health Sun et al.’s (2022). Furthermore, our study, consistent with Rahman et al.’s (2020) findings, revealed the importance of provider support in enhancing well-being.

The results of this study offer several implications for policymakers, especially concerning the challenges women encounter due to caregiving responsibilities, such as while pregnant (Pinnington et al., 2022). Enhancing women’s community embeddedness is critical and requires the formulation of strategic plans to achieve this objective. Therefore, we have formulated a list of recommendations for policymakers and practitioners:

First, policy makers should provide women with decision-making authority to promote their autonomy and empowerment, potentially improving their well-being and community embeddedness. Second, policymakers and NGOs in the UAE should enhance women’s access to education and economic opportunities, which can strengthen their integration within their communities. Third, women expatriates should be incentivized to actively pursue opportunities for community engagement, including joining social activities, volunteering, and participating in cultural events. Fourth, organizations and policymakers should develop more effective strategies to support women’s empowerment and community engagement by recognizing the significant link between relational empowerment and integration within a community. Fifth, to enhance well-being and community embeddedness, women should improve their capabilities and competencies, bolstering their ability to make informed decisions. Sixth, flexibility in work hours and locations should be allowed to demonstrate trust in employees and addresses their needs, particularly benefiting women who often balance multiple responsibilities. Implementing such policies can improve employee satisfaction and productivity. Seventh, work-life balance programs should be created as it helps women manage the demands of their work and family responsibilities (Saks, 2022). This can involve offering flexible work arrangements, providing access to resources that support family needs, and creating a work environment that supports women’s overall well-being. Eighth, established networking programs should be provided to women with guidance and connections that support their professional growth and community involvement. Policy makers should provide women with opportunities to leverage their networks for career advancement. This can include orientation workshops, mentoring programs, cross-functional projects, and job promotions that support both personal and professional growth (Pinnington et al., 2024). Finally, public awareness campaigns should be launched to highlight the importance of women’s contributions. The need for community support, in this aspect, can foster a more inclusive environment and encourage community embeddedness.

In our study, expatriates were grouped together as a single cohort to capture their broad expatriate experience in their host country of the UAE. This approach was chosen to provide an overview of common challenges and integration issues across different duration of residence in a host country. We recognize that the length of stay can influence an expatriate’s familiarity with the host culture and language, which may affect their community engagement and adaptation. To examine whether the length of stay in the UAE affected well-being and community embeddedness, we conducted an analysis of variance (ANOVA). The results indicated no significant differences in well-being and community embeddedness based on the number of years living in the UAE. The decision to group all expatriates together was based on the need to focus on overarching themes relevant to the expatriate experience, such as social integration and community engagement. This approach provided a general overview without introducing additional complexity related to the duration of stay. However, a previous study revealed that both career and community embeddedness are positively associated with the level of institutional trust experienced by self-initiated expatriates (Lehtonen et al., 2023).

## 6 Theoretical contribution

Empowerment theory provides a theoretical lens for this study to understand how expatriate women can overcome potential social and cultural barriers in host countries. The research highlights the relationship between relational empowerment and community embeddedness through well-being, enhancing our knowledge of how empowerment operates in multicultural settings. This extension of empowerment theory lays the groundwork for future research to explore the broader impacts of relational empowerment in other expatriate and international contexts. Furthermore, this research advances empowerment theory by emphasizing the importance of relational empowerment. The study enhances our understanding of community dynamics by examining how community settings influence individuals and the reciprocal interactions that occur as people exercise agency through their relationships in various contexts (Christens, 2012). Moreover, empowerment is not only an individual psychological state but is also significantly influenced by social relationships and community networks. By focusing on expatriate women, who often face unique barriers in diverse cultural settings like the UAE, this research provides valuable insights into the empowerment process.

Empowerment theory provides an effective framework for understanding how individual well-being relates to the broader social context, as discussed by Perkins and Zimmerman (1995), Zimmerman (1995). People with more relational empowerment are likely to be good at participating in both emotional and practical ways within the networks they are part of Christens (2012). Thus, this research additionally contributes to empowerment theory by demonstrating that the integration of women expatriates into a host society depends not on individual efforts, but on the relational empowerment they experience, which promotes both personal well-being and community embeddedness. This expands the application of empowerment theory to global and multicultural contexts, enriching its conceptual reach and practical relevance. Furthermore, this study advances the understanding of empowerment theory, particularly relational empowerment, by integrating it into the context of expatriate women’s well-being and community embeddedness in host countries. While previous researches have often focused on structural or individual empowerment, this study highlights how relational empowerment fosters well-being and community embeddedness.

Finally, this study contributes to the existing literature by expanding the concept of empowerment beyond the workplace or beyond gender equality discourses and into the expatriate domain. This is meant particularly for women living in culturally diverse and potentially challenging environment like the UAE. The study’s findings underscore that relational empowerment is not only critical for personal well-being but also serves as a pathway to greater community embeddedness.

## 7 Limitations and future studies

This study acknowledges several limitations. First, it concentrated only on women; future studies should include other genders. Second, the sample was from one country; future studies could include other countries (e.g., GCC). Third, this study relied on surveys and quantitative methods; future studies may conduct qualitative research using case studies and interviews to derive new insights into relational empowerment and community embeddedness among women expatriates. Fourth, the study relied on a cross-sectional design, which was used to investigate how women’s relational empowerment influences community embeddedness through well-being. To address this limitation, longitudinal designs are essential for tracking changes in these women’s relational empowerment over time and assessing its impact on both well-being and community embeddedness. Fifth, conducting comparative research across diverse cultural contexts could shed light on the interplay between cultural factors and community embeddedness, offering deeper insights into this phenomenon. Lastly, the use of convenience sampling means that the sample may not fully represent the broader population, potentially limiting the generalizability of the findings. The sample was predominantly composed of university students and individuals from specific groups with limited diversity in terms of socioeconomic status, religion, and occupation. Moreover, the survey did not collect detailed information on several factors such as participants’ salaries, family status, and specific job titles, which could impact their well-being and community embeddedness. Future research should address these limitations by incorporating a more diverse sample and including additional demographic variables such as income, family status, and employment positions. This would provide a more comprehensive understanding of how these factors influence well-being and community embeddedness.

## Data Availability

The original contributions presented in the study are included in the article/supplementary material, further inquiries can be directed to the corresponding author.

## Appendix

Wellbeing taken from Topp et al. (2015).

Over the past 2 weeks

1. I have felt cheerful and in good spirits
2. I have felt calm and relaxed
3. I have felt active and vigorous
4. I woke up feeling fresh and rested
5. my daily life has been filled with things that interest me

Relational Empowerment taken from Peterson et al. (2021).

1. I work to build common cause with people who are different from me
2. I work to build common cause among people who are different from each other
3. I bring people from different backgrounds together to achieve a common goal
4. I try to help others that are facing the same challenges I have faced
5. I connect others to resources and opportunities that can benefit them
6. I encourage others to get involved in community activities
7. I help and support other people who are trying to make changes in their lives
8. I try to connect people whose experiences with recovery can benefit others
9. I share what I have learned about recovery with others

Community embeddedness taken from Felps et al. (2009).

1. I really love the place where I live.
2. The place where I live is a good match for me.
3. The area where I live offers the leisure activities that I like (sports, outdoor activities, cultural events & arts).
4. Leaving the community where I live would be very hard
5. If I were to leave the area where I live, I would miss my neighborhood
6. I am a member of an effective work group.
7. My family roots are in this community.
8. I am active in one or more community organizations (e.g., churches, mosque, sports teams, schools, etc.)
9. I participate in cultural and recreational activities in my local area
